# Cutting-Edge AI Technologies Meet Precision Medicine to Improve Cancer Care

**DOI:** 10.3390/biom12081133

**Published:** 2022-08-17

**Authors:** Peng-Chan Lin, Yi-Shan Tsai, Yu-Min Yeh, Meng-Ru Shen

**Affiliations:** 1 Department of Oncology, National Cheng Kung University Hospital, College of Medicine, National Cheng Kung University, Tainan 704, Taiwan; 2 Department of Genomic Medicine, National Cheng Kung University Hospital, College of Medicine, National Cheng Kung University, Tainan 704, Taiwan; 3 Department of Medical Imaging, National Cheng Kung University Hospital, College of Medicine, National Cheng Kung University, Tainan 704, Taiwan; 4 Institute of Clinical Medicine, National Cheng Kung University Hospital, College of Medicine, National Cheng Kung University, Tainan 704, Taiwan; 5 Department of Obstetrics and Gynecology, National Cheng Kung University Hospital, College of Medicine, National Cheng Kung University, Tainan 704, Taiwan; 6 Department of Pharmacology, National Cheng Kung University Hospital, College of Medicine, National Cheng Kung University, Tainan 704, Taiwan

**Keywords:** artificial intelligence, bioinformatics, next-generation sequencing, high-performance computing, precision medicine, cancer genomics

## Abstract

To provide precision medicine for better cancer care, researchers must work on clinical patient data, such as electronic medical records, physiological measurements, biochemistry, computerized tomography scans, digital pathology, and the genetic landscape of cancer tissue. To interpret big biodata in cancer genomics, an operational flow based on artificial intelligence (AI) models and medical management platforms with high-performance computing must be set up for precision cancer genomics in clinical practice. To work in the fast-evolving fields of patient care, clinical diagnostics, and therapeutic services, clinicians must understand the fundamentals of the AI tool approach. Therefore, the present article covers the following four themes: (i) computational prediction of pathogenic variants of cancer susceptibility genes; (ii) AI model for mutational analysis; (iii) single-cell genomics and computational biology; (iv) text mining for identifying gene targets in cancer; and (v) the NVIDIA graphics processing units, DRAGEN field programmable gate arrays systems and AI medical cloud platforms in clinical next-generation sequencing laboratories. Based on AI medical platforms and visualization, large amounts of clinical biodata can be rapidly copied and understood using an AI pipeline. The use of innovative AI technologies can deliver more accurate and rapid cancer therapy targets.

## 1. Introduction

**Figure 1:**
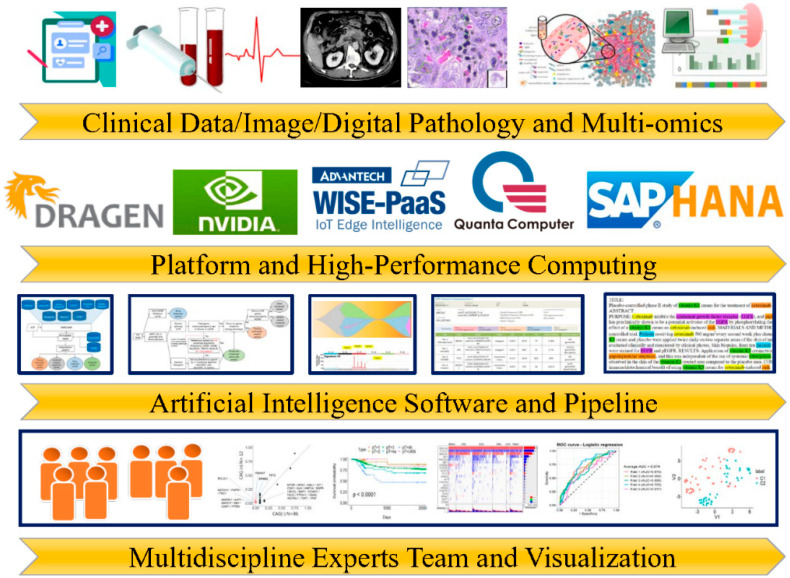
**Clinical practice for precision cancer genomics and artificial intelligence-powered bioinformatic technologies.** Artificial intelligence techniques, software platforms, and high-performance computation have been used extensively to provide improved cancer care via clinical patient data, such as electronic medical records, physiological measurements, biochemistry, computerized tomography scans, digital pathology, and the genetic landscape of cancer tissue.

Artificial intelligence (AI) techniques, platforms, and high-performance computation have been extensively used in cancer genomics precision medicine. Many sources of clinical data of cancer patients exist, such as electronic medical records, physiological measurements, biochemistry, computerized tomography scans, digital pathology, and the genetic landscape of cancer tissue for clinical diagnosis, treatment, and monitoring (Figure 1). Many AI medical platforms, such as NVIDIA, QOCA (Quanta computer incorporated), Advantech, and system analysis program (SAP)/high-performance analytic appliance (HANA), can serve as big biodata sources. Physicians can quickly copy and understand large amounts of clinical biodata by using high-performance computing (HPC) and AI pipelines. Multidisciplinary teams of professionals, such as physicians, biostatisticians, and information (IT) technicians, are needed to establish operational flows. This article discusses the use of AI techniques in cancer genomics based on high-performance computing.

In cancer patients, many bioinformatic analysis workflows are used to investigate cancer targets and monitor strategies using DNA whole-genome/whole-exome sequencing (WGS/WES) and circulating tumor DNA, RNA, and tumor deep-targeted sequencing (1). In addition to the use of genetic changes, the clinical use of a tumor mutation burden (TMB), microsatellite instability (MSI), and mutational signature patterns in cancers was reported by Bødker et al. [1]. We previously created an in-house bioinformatic pipeline for data processing and analysis [2]. The Cancer Next-Generation Sequencing (NGS) Laboratory at the National Cheng Kung University Hospital (NCKUH is a hospital in North District, Tainan, Taiwan) provided the genome analysis workflow. DNA was extracted from the blood and tumor tissues of cancer patients and sequenced using next-generation sequencers, such as the Illumina sequencing and Oxford Nanopore systems. We used HPC systems, such as NVIDIA graphics processing units (GPUs) and DRAGEN field programmable gate arrays (FPGAs) systems, to accelerate genomic analysis.

This article reviews new AI technologies for genome analysis, focusing on four primary issues. First, we examine the computational prediction of pathogenic variants and create a pipeline for the interpretation of genetic mutations regarding clinical cancer susceptibility [3]. Second, new cancer therapy options, such as the analysis of somatic genetic mutations, mutational signatures, and cancer evolution, have been developed using AI models. Third, we review the single-cell genomics sequencing technologies and computational techniques in cancer biology. Fourth, we discuss text-mining, which offers a practical approach for identifying cancer gene targets. Finally, to improve performance, we examine how clinical NGS laboratories deploy HPC via NVIDIA, DRAGEN systems, and AI medical cloud platforms. After visualizing and integrating a data-management system, AI has introduced new concepts and clinical practices focusing on genomics precision medicine. The use of advanced AI technologies can facilitate the more precise and rapid identification of potential cancer-treatment targets.

## 2. Computational Prediction of Pathogenic Variants of Cancer Susceptibility Genes

Owing to the genome sequencing of germline mutations, the clinical diagnosis of hereditary genetic cancer patients has been translated into treatment options. Immunotherapy and poly (ADP-ribose) polymerase inhibitors are used as first-line treatments in colorectal and ovarian cancers [4,5]. Germline genetic variations may contribute to carcinogenesis, therapeutic efficacy, toxicity, and cancer phenotype. It remains difficult for clinicians to interpret the influence of these mutations on distinct phenotypes.

A diverse range of AI-based diagnostic tools has been developed using various computational genomic models. The American College of Medical Genetics and Genomics and the Association for Molecular Pathology (ACMG/AMP) have published guidelines for interpreting sequence variations [6]. Multiple prediction scores can be identified and segregated for missense variants. The ACMG refers to three different prediction algorithms, namely, the Rare Exome Variant Ensemble Learner (REVEL) ensemble method, based on combinations of scores; genetic conservation site predictors [7]; and splicing site predictions. The application of existing silicon models to evaluate pathogenic variants in a particular gene, including alternative splicing with protein disruption, is greatly limited. As a result, we have divided our algorithm into three categories: sequencing-based, amino-acid/protein-based, or ACMG-criteria-provided-based. Table 1 lists AI-based genetic variations for which machine learning methods can be used to estimate pathogenicity. The scoring features and sources of the in silico prediction tools are also categorized.

### 2.1. Sequencing-Based Prediction

Sequence-based techniques [8,9,10,11,12,13,14,15,16,17,18] are used to construct the most common tools for predicting the pathogenicity of genetic variations. For instance, REVEL [10] employs a random forest method based on ensemble methods with 13 pathogenicity predictors. REVEL scores can identify human pathogenic missense mutations and rare variants. CADD [12] is another ensemble method that integrates several scoring algorithms using a linear kernel support vector machine. VEST4 [8], EvoDiagnostics [18], and MetaSVM [9] are well-known random forest (RF) and support vector machine (SVM) prediction tools. Random forests and SVM may handle linear and non-linear data. The random forests classifier is an ensemble of various distinct decision trees (uncorrelated models). It will outperform each of its individual models. ACMG uses REVEL, random forests, for ensemble computational prediction models. SVM is a supervised learning model for regression analysis and non-probabilistic binary linear classifiers. The algorithms can evaluate genomic data for pathogenic classification. Another ensemble technique is CADD. To increase the forecast accuracy for various numerical pathogenetic scoring tools, CADD uses the ensemble regression technique to aggregate many models.

Artificial neural networks, such as convolutional neural networks (CNN) and ResNets, have been employed by Primate AI [11] and missense variant pathogenicity prediction (MVP) [16] to predict the pathogenicity of missense variations. Splice AI [13] can accurately predict splice junctions from an arbitrary pre-mRNA transcript sequence based on a deep neural network (DNN). Other novel prediction tools based on the utilized recurrent neural network, XGBoost (a variant of the gradient boosted tree), include 3Cnet [14] and VARITY [17]. The neural network can manage large training datasets and many correlated predictors. The parameters of the aforementioned distribution are modeled by a neural network when trained for a classification or regression task. For example, MVP, based on ResNets, could better prioritize pathogenic missense variants, especially in de novo genetic mutations and tolerance loss of function genetic variants. Splice AI estimates more than 10% of the pathogenic mutations, previously unrecognized mutations, in patients with rare genetic illnesses.

Constructing an AI algorithm to predict phenotype-specific genetic variations remains challenging. We previously developed a matrix factorization-based algorithm to predict the phenotype of chemotherapy-induced neuropathy. The matrix factorization method was evaluated using The Cancer Genome Atlas (TCGA) patient WES data and showed an improved performance (accuracy > 98%) [15].

### 2.2. Amino-Acid- or Protein-Based Prediction

Amino-acid- or protein-based [19,20,21,22,23,24] pathogenicity prediction approaches are promising because they consider the context of amino acid sequences and minimize overfitting to prior sequencing-based knowledge. Therefore, it is critical to create single amino-acid- or protein-based prediction models. AI technologies have also been used to predict protein function. For example, PROVEAN [19] predicts pathogenicity using a delta alignment score. Another ensemble method is ProtVec [20], which incorporates natural language processing (NLP) with support vector machines. The authors distribute the representation of biological sequences using the NLP techniques. BioSeq-Analysis 2.0 [21] and Rhapsody [22] are well-known random forest tools. BioSeq-Analysis 2.0 includes a classification algorithm modified from LIBSVM and a sequence-labeling algorithm based on conditional random fields. LYRUS [23] is a new prediction tool, developed by XGBoost (a highly efficient gradient-boosting decision tree, GBDT). We also previously developed a single amino acid variant of the Light Gradient Boosting Machine (LightGBM) [24] based on protein structural energies. Compared with the sequencing-based AI model, amino acid/protein-based techniques use a different database, such as Protein Data Banks (PDBs) or the Database of Protein Disorder (DisProt). Data preprocessing is another critical issue for the prediction model. For example, we use the PDBs and Rosetta energy function for pathogenic prediction [24].

### 2.3. AI Tools Based on ACMG/AMP and Functional Somatic Mutation

Traditional AI in bioinformatics uses sequence alignment matching, protein–protein interactions, and structure–function analysis to assist in cancer genome research. Such research will aid in the development of molecularly targeted medications. Several cancer drugs that directly target genetic alterations have been evaluated in clinical trials. For example, osimertinib can be used to treat individuals with non-small cell lung cancer (NSCLC) harboring the T790M EGFR mutation [29]. However, cancer is a genetically heterogeneous illness. Therapeutic targets could include somatic genetic mutations, mutational signatures, and cancer evolution. Table 1 shows ACMG-based genetic pathogenic mutation prediction and AI models for mutational signatures and cancer evolution. This section covers AI methods for ACMG prediction tools and the implications of mutational signatures and tumor evolution for drug development, including strategies to lower the likelihood of drug resistance. The classical AI classifier quantitates potentially damaging genes; they did not consider the pathogenicity evaluation in a disease. ACMG guidelines include disease information, such as population allele frequency, functional data of mutation, and segregation analysis in families, to evaluate the pathogenicity of genetic mutations.

For the pathogenicity interpretation of germline variants, the ACMG and the United Kingdom (UK) Association for Clinical Genomic Science (UK-ACGS) both provide updated consensus criteria for the evaluation and classification of pathogenic variations [6,30]. The policy includes 28 attributes with codes addressing different types of evidence to navigate the clinical interpretation of rare diseases. Each variant is assigned a pathogenicity assertion depending on the criteria used. In ClinVar [31], a public dataset, variants are classified as pathogenic, likely pathogenic, having unclear significance, likely benign, or benign, based on review status. The developed machine-learning algorithms were assessed in terms of classification and prioritization. Standard tools for interpreting pathogenicity based on ACMG criteria include modelling ACMG/AMP [25], CharGer [26], VarSome [27], and Clinvitae [28] (Table 1). CharGer [25] and VarSome [27] assign scores to each genetic variant depending on the amount and strength of the evidence provided by the ACMG/AMP criteria. Tavtigian et al. [25] developed a model of ACMG scores using a Bayesian classification system. Clinvitae et al. [28] used penalized logistic regression to prioritize and classify the pathogenicity of genetic variants. We also created an ACMG/AMP-based score computation and designed unique algorithms to assign a score or degree of pathogenicity [14].

A four-tiered framework has been presented for cancer somatic mutations to characterize somatic sequence variants depending on their clinical importance [32]. In somatic mutations, variants in genes associated with pathogenicity have been characterized. Many cancer-variant databases are available, including the Cancer Genome Interpreter (CGI) [33], Clinical Interpretation of Variants in Cancer (CIViC) [34], the Jackson Laboratory Clinical Knowledgebase (JAX-CKB) [35], OncoKB [36], and the Precision Medicine Knowledgebase (PMKB) [37], which have been used to interpret somatic variants of cancer. Many tools have been developed for modeling somatic mutations, such as MuSE, MuTect, SomaticSniper, Strelka, and VarScan2 [38]. Ng et al. developed a functional genomics platform (FASMIC) [39] to identify the driver mutations for potential clinically actionable genes. FunSeq2 is a tool used for prioritizing and annotating non-coding somatic variants [40]. However, many laboratories have reported functional somatic mutations based on ACMG/AMP criteria.

## 3. AI model for Mutational Analysis

### 3.1. Mutational Signatures and AI Tools

To investigate somatic genetic mutagenesis, mutational signatures have revealed the probable mechanisms of cancer etiology and biological processes. The mutational signature landscape can potentially indicate drug-responsive and resistance biomarkers and prognostic factors for cancer. For example, the sensitivity of ovarian cancer to poly (ADP)-ribose polymerase (PARP) inhibitors is linked to mutational signatures related to homologous recombination deficiency. In contrast, APOBEC-related mutational signatures are associated with responses to ataxia telangiectasia and Rad3-related kinase (ATR) inhibitors. Our previous studies found distinct characteristics of mutational signatures in patients with cancer-associated genetic variations. These mutational signatures provide additional information on the etiology and progression of individual cancers, as well as new biomarkers for cancer treatment [41]. New technologies and machine learning algorithms have increased the feasibility of identifying mutational signatures [42,43,44,45,46] and facilitated the integration of signature analysis into clinical decision-making. The Catalogue of Somatic Mutations in Cancer (COSMIC) Signatures [42], DeaminationSigs [44], and SparseSignatures [45] are standard tools for cancer etiology and quality control based on non-negative matrix factorization (NMF) algorithms (Table 2). For DeconstructSigs, a multiple linear regression model was used [43]. Chevalier et al. established an analysis and visualization tool to characterize and enhance the discovery of mutational signatures [46].

### 3.2. Cancer Evolution and AI Tools

Genetic mutations are characterized by a somatic evolutionary process that contributes to cancer development, progression, and drug resistance. At present, many algorithms are available for the analysis of clonal evolution [47,48,49,50,51,52]. To solve the problem of intra-tumor heterogeneity (ITH) from bulk DNA sequencing, numerous computational methodologies and instruments have been developed which analyze genome data to reconstruct and describe the clonal evolutionary landscape. There are three prominent roles for the clonal evolutionary development of cancer: clustering genetic variants by cell fraction, reconstructing the cancer clonal evolution tree, and visualizing clonal extension. DeCiFering [52] and ClonEvol [50] were designed for genetic variant clustering based on the descendant cell fraction and bootstrap resampling. LICHeE [47], SCHISM [48], and Canopy [49] used directed acyclic graphs (DAGs).and Bayesian methods to reconstruct phylogeny. PACTION [51] is a straightforward and rapid strategy that reconstructs the clonal architecture of cancer tumors based on mixed-integer linear programming (MILP). Fish plots and timescapes have been used to visualize the evolution of cancer. For the development of cancer therapeutic targets, our study identified genetic subclones, and clusters were identified using SciClone [53] based on a Bayesian clustering method. ClonEvol has been used to visualize the evolution of somatic mutations in cancers. We highlight the significance of cancer evolution models in the development of new methodologies for drug targets.

### 3.3. Clinical Practice in Mutational Signature and Cancer Evolution

Mutational signatures reveal information about mutagenic processes in cancer patients and the quality of genetic mutation detection in cancer tissues. In addition to homologous recombination deficiency (mutational signature SBS3) and APOBEC-mutagenesis (mutational signature SBS2), we could detect mutational signatures SBS6, SBS14, SBS15, SBS20, SBS2, SBS26, and SBS44 in cancer patients with mismatch repair deficiencies [41,42]. These could potentially be used as immunotherapy biomarkers. In cancer tissues fixed in formalin, the mutation signature SBS1 was increased (spontaneous deamination of methylated cytosine). To reduce the impact of deamination, a mutational signature analysis should be considered in routine quality reports.

Cancer evolution can reveal the clonal nature of the driver mutations in the evolution process. It can also guide therapy by focusing on clonal and subclonal genetic mutations. Patients with cancer recurrence or drug resistance may benefit from an analysis of the cancer’s evolution over time. For example, the *BRAF* clonal mutation remains resistant to *BRAF* inhibitors in some melanomas with co-existing alterations to other clonal genetic mutations. Using the cancer evolution model, we found that concurrent sequential *BRAF* mutations also affected hypermutation status. Compared to *AKT*-*BRAF* sequential mutations, *PETN*-*BRAF* sequential mutations were significantly more frequent in hypermutated cancers. The cancer evolution model may guide clinical practice. We also built a cancer evolution model based on the NGS data and applied machine learning analysis to identify potential evaluation therapeutic targets, such as DNA repair, *MYO18A*, and *FBXW7* genetic mutations in CRCs [54]. Using an AI model for mutational analysis could provide us with more detailed clinical cancer information.

## 4. Single-Cell Genomics and Computational Biology

Among the single-cell genomics technologies, epigenome sequencing, genome sequencing for lineage tracking, spatially resolved transcriptomics, and omics sequencing are the newest developments. Data from single-cell genomics are sparse and high-dimensional, which makes machine-learning analysis challenging. The high-dimensional data were typically reduced using principal component analysis (PCA), t-distributed stochastic neighbor embedding (t-SNE), and uniform manifold approximation and projection (UMAP). Table 3 shows the AI methods for computational biology in signal-cell genomics, including omics data integration, cell type classification, and trajectory inferences [55,56,57,58,59,60,61,62,63,64,65,66,67].

MOFA+ is a statistical framework for data integration. It reconstructs a low-dimensional data representation using stochastic variational inference that is amenable to GPU computations [55]. Sparse canonical correlation analysis (sCCA) also computed sparse latent variables to predict complex traits [56]. These AI models support flexible sparsity data management in the same way as Penalized Integrating Matrix Factorization (PIntMF) [59]. Cao et al. use unsupervised topological alignment for single-cell multi-omics integration [57]. A graph convolutional networks algorithm was used to integrate disparate and interaction datasets [58]. Two examples were shown for cell type classification. The ACTINN [60] and Ikarus [61] used a neural network or logistic regression model to distinguish the immune cell or tumor cell.

The developmental trajectories could be computationally inferred using trajectory inference algorithms in single-cell genomics. CellRouter [62] uses tree methods to model the trajectory based on the context likelihood of relatedness. The STREAM [63] and TinGA [64] used graph methods for trajectories based on the gaussian process latent variable model or growing neural graph algorithm, respectively. The ELPIgraphy [65] used cyclic methods for trajectories based on elastic energy functional and topological graphs. CStreet also used the k-nearest neighbor graph for trajectories [66]. Tenha et al. [67] use Euclidean minimum spanning tree methods to model the trajectory based on single-cell biology. Based on the computational biology of signal cell techniques, we accelerated the identification of new cancer cell types and understood the disease trajectories. This may help us to define new cancer subtypes and monitor therapeutic responses.

## 5. Text Mining for Identifying Genes Targets in Cancers

The biomedical literature has presented far-reaching findings for drug-target identification and cancer treatment, including their biological significance (molecular and cell activities and signal pathways). Data mining is a machine-learning technique that works with AI technologies or statistical methods to identify optimal cancer targets in biomedical science. The link between disease and genetic alterations is critical to obtaining a better understanding of cancer biological mechanisms. Gene2Vec, a study that explored the idea of gene embedding in the spirit of word embedding, is one of the new forms of text-mining [68]. However, we could not explain the biological significance of the vector in the neural-embedding model. Table 4 [69,70,71,72,73,74] shows that various data-mining techniques have been used to classify gene-mutation diseases.

Several text-mining methods have been developed for mutation–disease relationships. For example, MuGeX [69] employs the Nave Bayes/Rocchio algorithm-IDF to retrieve mutation–gene combinations from Medline abstracts in response to a disease query. tmVar 2.0 [72] is an approach that integrates genetic variant information from the literature with the Single Nucleotide Polymorphism Database (dbSNP) and ClinVar using conditional random fields (CRF). Several ML classifiers were tested, such as the C4.5, decision tree, multilayer perceptron, and Bayesian logistic regression. Singhal et al. [70] employed the C4.5 decision tree to create an automated pipeline that uses the full-text biomedical literature and is validated using evidence-based gene panels. This approach is focused on disease–mutation relationships. To infer variant-driven gene panels, Saberian et al. [73] integrated GNormPlus [75], tmVar 2.0 [72], and DNorm [76] into MAGPEL for variant–genotype–phenotype prediction. EnzyMiner [71] used probabilistic indexing for protein mutation prediction, automatically identifying information on protein stability or enzyme activity from PubMed abstracts.

Our own work contextualized the genes for clinical precision medicine, presenting druggable targets, hereditary cancer syndrome mutations, and illness subgroups [74]. The hypergeometric test was used to construct the mutational landscape of the actionable cancer genome from the biomedical literature, which was then confirmed using the NGS database. Our platform may enable the development of a cancer gene panel recommendation system for precise cancer therapy.

## 6. The NVIDIA GPUs, DRAGEN FPGAs Systems, and AI Medical Cloud Platforms in the Clinical NGS Lab

### 6.1. Using NVIDIA GPUs, DRAGEN FPGAs Systems in Bioinformatic Analysis

Recent developments in HPC and biological data analysis technologies have resulted in the rapid growth in biological analyses. The use of HPC in bioinformatic analysis enables the efficient processing of large amounts of data in everyday clinical practice. We previously used hardware accelerators, such as GPUs and FPGAs, to speed up and maximize throughput of cancer genomics. In clinical services, we utilized the NVIDIA Parabricks and Illumina dynamic read analysis for genomics (DRAGEN) platforms (Table 5) [77,78,79,80].

The NVIDIA Parabricks (GPUs) software suite analyzes whole-genome and exome sequencing data. It significantly improves throughput times for common genomic investigations, such as germline and somatic research. The NVIDIA Clara Parabricks toolkit includes germline Deepvariants, somatic, RNA, and human population pipelines. Precise and clear results were obtained for RNA analysis. A Signature Analyzer-GPUs has been used for mutational signature analysis [77]. Haradhvala et al. discovered mutational signatures linked to loss of POLE proofreading and mismatch repair [77]. Such information may help to inform clinical decisions concerning immunotherapy targets for cancer treatment. Gorzynski et al. [78] connected long-read sequencing (nanopore technology) and GPUs in an acute scenario to enable the real-time analysis of ultrarapids. GPUs-accelerated tools on NVIDIA Clara Parabricks pipelines for cancer and germline analyses are helpful in clinical situations.

The Illumina DRAGEN Bio-IT Platform (FPGAs) enables precise, comprehensive, and rapid analyzes of NGS data. Updated algorithms for genetic data analysis can be provided using FPGAs-based bioinformatic acceleration devices. TruSight Oncology 500 Assay tumor profiling [79] and liquid biopsy NGS [80], analyzed using FPGAs, were recently developed for cancer treatment-monitoring techniques. These utilize tumor mutation burden, microsatellite instability, and genetic alteration information for cancer diagnosis, prognosis, and treatment. They will also provide for practical use of the homologous recombination deficiency (HRD) score in future treatment of ovarian cancer. We built a workflow in NCKUH to speed up the study of the complete exosome genome and tumor deep-target sequencing for clinical cancer management. These tools can be used for reliable and timely genetic diagnostics (Figure 1).

O’Connell et al. benchmarked two germline variant callers and four somatic variant callers. They compared traditional x86 CPU algorithms with GPU-accelerated algorithms implemented with NVIDIA Parabricks on Amazon Web Services (AWS) and Google Cloud Platform (GCP). For germline callers, the author observed speedups of up to 65× (GATK haplotype caller). Alternatively, somatic variant callers achieved speedups of up to 56.8× (Mutect2 algorithm) [81]. For emergency use for hospitalized patients, Clark et al. built a pipeline based on the DRAGEN platform to analyze genome sequencing data. A median delivery time of less than 24 h was observed from blood samples to provisional findings. High accuracy and sensitivity were also observed [82].

### 6.2. AI Medical Cloud Platforms for Cancer Care

Many AI medical cloud platforms have been developed, including QOCA, Advantech, and SAP/HANA, that could integrate AI technologies for genome visualization and precision medicine for cancer care. In cancer clinics, medical imaging data must be integrated to visualize genomic data, select cancer-target drugs, and predict cancer survival. Integration of the system analysis program (SAP) cloud platform for genomic and clinical data enables practitioners to quickly evaluate and make sense of the data. The Variant Browser visualizes genetic variations and integrates patient clinical data stored in a clinical data warehouse as variant information, as well as genomic interactions relating to specific patients.

The Advantech edge visualization solution efficiently iterates using data-intensive visualizations. We previously collaborated with Advantech to create a cancer clinic dashboard that visualizes multiomics data and clinical information. Real-time recommendations could be provided to patients using advanced platform and visualization technology. QOCA is an AI-assisted platform for medical imaging and autonomous inference; it now plays an essential role in intelligent medical solutions. For example, by using QOCA electrocardiography (ECG) monitoring devices, clinicians might effectively monitor cancer patients receiving portable cancer treatment at home.

For the integration of multi-omics and medical images, we recently demonstrated that utilization of the covariate-adjusted tensor classification in the high-dimensional (CATCH) model could accurately classify recurrent colorectal cancer by combining adjusted radiomics-based CT images with RNA immune genome expression data. We integrated medical images and genome data into an operational flow for recurrent stage III colorectal cancer and provided individualized treatment strategies [83].

## 7. Conclusions

Five main viewpoints should be considered when providing precision medicine and cancer care. Before treatment, it is essential to identify inherited cancers with (i) cancer susceptibility genes and (ii) AI models for mutation analysis. For example, we identified MLH1 germline genetic mutations based on whole-genome sequencing in colorectal cancer patients [3]. For therapeutic strategies, immunotherapy, instead of traditional chemotherapy, may be the first choice for first-line treatment in metastatic colorectal cancer patients [4]. Another example is a cancer patient with cardiovascular *KCNH2* genetic variants; EKG showed the QTc prolonged during the chemotherapy. For better cancer care, we should carefully monitor the EKG during chemotherapy if the patient carries *KCNH2* genetic variants. (iii) Single-cell genomics may provide data for disease surveillance. We can suggest cancer gene panels to patients based on (iv) text-mining findings for cancer patients with refractory treatment. Mutation analysis, such as somatic mutation, mutational signature, and cancer evolution, could provide therapeutic strategies or targets for cancer patients. The intelligent hospital must set up (iv) telemedicine devices and high-performance computing for real-time, in-person patient care.

For high-risk stage II and stage III colorectal cancer patients, NCKUH developed an AI precision medicine platform to manage Big Biodata. We developed an AI tracking and alarm system for the electronic medical record, biochemistry data, genetics, and CT scan image analysis to improve survival and quality of life. We demonstrated that germline susceptibility and deletion structural variants can have an impact on the survival and therapeutic strategies for stage III colorectal cancer. For example, patients with germline DNA repair genetic variants and *CEP72* deletion structural variants have better survival in CRCs [84]. Using AI model analysis, we could stratify the risk of cancer recurrence. For the application of the cancer evolutional model, we identify potential evaluation therapeutic targets, such as *MYO18A*, and *FBXW7* genetic mutations in CRCs [54]. To determine the oncology image biomarker, we integrated adjusted CT images into genome data to accurately classify recurrent CRC. We could use this AI model to provide individualized cancer therapeutic strategies based on adjusted radiomic features in recurrent stage III CRC [83]. To improve the long-term quality of life, we will establish the AI model to predict chemotherapy side-effects, such as neuropathy and sarcopenia, for CRCs in the future.

Cancer care can be provided via telemedicine using AI-based technology. For example, the QOCA provides the AI medical cloud platform (QOCA aim), AI health care platform (QOCA apc), and AI telemedicine platform (QOCA atm). AI medical cloud platform (QOCA aim) can provide the best clinical decision assistance and accurate AI prediction with medical images and structured data analysis. For example, we established the AI model to predict cancer recurrence in stage II and III CRCs via standardized pathology reports. This can be a powerful tool for sharing the decision-making between physicians and patients. The QOCA apc, a case manager, provides a platform including the daily activities and physiological monitors, such as the heart rate, O2 saturation, body temperature, and glucose levels of cancer patients. Cancer patients who received home-based chemotherapy can be closely monitored via QOCA apc. QOCA atm, a hospital-to-home platform, could help us manage patient needs, such as nutrition supplements and the adverse effects of chemotherapy, via real-time face-to-face interaction.

Choosing the proper service model at the end of life (EOL) for physicians and patients, such as hospice share care (HSC), hospice inpatient care (HIC), and hospice home care (HHC), is a typical challenge. For hospice home care, we set up an artificial intelligence services platform and built an AI model to suggest a services model based on the patient’s characteristics, symptoms, and hospice care needs [85]. Our artificial intelligence hospice services deliver goal-directed care to alleviate symptoms and provide holistic care to terminal patients. We installed a computerized detection system for hospice home care with the QOCA apc. We identify the health conditions of patients in the end-of-life period using the EKG and O2 sensing system. We applied QOCA atm to home-based hospice and palliative care. We could watch the patients and easily inform their family about their terminal status early in the process. We could deliver personalized end-of-life care and reduce the burden of care for family members through telemedicine and AI technology. With intelligent remote medicine technology, we can improve quality of life for terminal patients and respond to the core value of the need for dignity while dying at home.

The extensive use of WGS/WES has completely changed the diagnostic procedures in medical genetics, particularly for cancer, non-invasive prenatal screening, childhood development, and rare disorders. The incidence of cancer has dramatically increased over the last decade. WGS/WES in germline and somatic mutations can provide cancer diagnosis and the etiology of cancer. For example, the mutational signature of urothelial carcinoma showed that aristolochic acid exposure plays a vital role in Taiwan [86]. This could be a screening tool for cancer etiology to determine the public health policy. Concerning public health issues, we can screen for cancer, create a preventive procedure for cancer, and promote lifestyle changes in inherited or high-risk cancer populations [87]. Contrary to the single or multiple gene panels, we performed WGS/WES-based genomic analyses using AI-based high-performance computing methods. More quick and accurate diagnoses could be reached. Pharmacological side-effects (pharmacogenomics) and the ACMG non-oncogenic phenotype are other significant public health issues, particularly for cancer patients. Implementing AI-based algorithms in high-performance computing is most urgent due to the public health concerns.

AI-powered analytical techniques with HPC platforms are widely used in clinical practice for precision cancer genomics. Clinicians must grasp the concept of big data within the healthcare field and translate it into usable knowledge for real-time patient care. Future research should, therefore, focus on the fast-evolving fields of bioinformatics, AI medical clouds, and visualization platforms. AI-powered bioinformatics technologies are expected to routinely provide clinical diagnostic and treatment services in the future.

## Figures and Tables

**Table 1:** AI-based prediction models for the pathogenicity of genetic variants.

Methods	Categorical Prediction	Algorithms	Author
**Sequencing-based prediction**			
VEST4	Higher scores are more deleterious	RF	Carter et al., 2013 [8]
MetaSVM	Higher scores are more deleterious	Radial kernel SVM	Dong et al., 2015 [9]
REVEL	Higher scores are more deleterious	Ensemble methods/RF	Ioannidis et al., 2016 [10]
Primate AI	Higher scores are more deleterious	Convolutional neural network	Sundaram et al., 2018 [11]
CADD	Higher scores are more deleterious	Linear kernel SVM	Rentzsch et al., 2019 [12]
Splice AI	Higher scores are more deleterious	Deep neural network	Jaganathanet al., 2019 [13]
3Cnet	Higher scores are more deleterious	Recurrent neural network	Won et al., 2021 [14]
CoLaSp	Higher scores are more deleterious	Latent space matrix factorization	Abdollahi et al., 2021 [15]
MVP	Higher scores are more deleterious	ResNets	Qi et al., 2021 [16]
VARITY	P: Pathogenicity; B: Benign	XGBoost	Wu et al., 2021 [17]
EvoDiagnostics	P: Pathogenicity; B: Benign	RF	Labes et al., 2022 [18]
**Amino acid or protein-based prediction**			
PROVEAN	D: Deleterious; N: Neutral	Delta alignment score	Choi et al., 2015 [19]
ProtVec	P: Pathogenicity; B: Benign	NLP/SVM	Asgari et al., 2015 [20]
BioSeq-Analysis 2.0	P: Pathogenicity; B: Benign	RF/SVM	Liu et al., 2019 [21]
Rhapsody	Pathogenicity probability	RF	Ponzoni et al., 2020 [22]
LYRUS	P: Pathogenicity; B: Benign	XGBoost	Lai et al., 2021 [23]
LightGBM	P: Pathogenicity; B: Benign	LightGBM	Wu et al., 2021 [24]
**ACMG/AMP-based model**			
Modelling ACMG	P: Pathogenicity; B: Benign	Bayesian classification framework	Tavtigian et al., 2018 [25]
CharGer	Higher scores are more deleterious	Databases and criteria-based	Scott et al., 2019 [26]
VarSome	P: Pathogenicity; B: Benign	Databases and criteria-based	Kopanos et al., 2019 [27]
Clinvitae	P: Pathogenicity; B: Benign	Penalized logistic regression	Nicora et al., 2022 [28]

Notes: RF: random forest; SVM: support vector machine; and NLP: natural language processing.

**Table 2:** AI tools in bioinformatics for mutational analysis.

Methods	DATA	Algorithms	Author
**Mutational signatures**			
COSMIC Signatures	SNV/indels	Non-negative matrix factorization	Alexandrov et al., 2020 [42]
DeconstructSigs	SNV/indels	Multiple linear regression model	Rosenthal et al., 2016 [43]
DeaminationSigs	SNV/indels	Non-negative matrix factorization	Bhagwate et al., 2019 [44]
SparseSignatures	SNV	Non-negative matrix factorization	Lal et al., 2021 [45]
Musicatk	SNV	Non-negative matrix factorization/LDA	Chevalier et al., 2021 [46]
**Tumor evolution model**			
LICHeE	SNV/CNV	Directed acyclic graph	Popic et al., 2015 [47]
SCHISM	SNV/CNV	Directed acyclic graph	Niknafs et al., 2015 [48]
Canopy	SNV/CNV	Bayesian mixture models	Jiang et al., 2016 [49]
ClonEvol	SNV/CNV	Bootstrap resampling	Dang et al., 2017 [50]
PACTION	SNV/CNV	Mixed integer linear programming	Sashittal et al., 2022 [51]
DeCiFering	SNV	Descendant cell fraction	Satas et al., 2022 [52]

Notes: ACMG, American College of Medical Genetics and Genomics; SNV, single-nucleotide variant; CNV, copy number variations; and LDA, latent Dirichlet allocation.

**Table 3:** Single-cell genomics and computational biology.

Methods	Goal	Algorithms	Author
**Single-Cell Omics Data Integration**			
MOFA+	Sparse data	Stochastic version of the algorithm	Argelaguet et al., 2020 [55]
sCCA	Sparse data	Sparse canonical correlation analysis (CCA)	Rodosthenous et al., 2020 [56]
Unicom	Distance matrix	Unsupervised topological alignment	Cao et al., 2020 [57]
sGCN	High-dimensional data	Graph convolutional networks	Song et al., 2020 [58]
PIntMF	Sparse data	Penalized integrative matrix factorization	Pierre-Jean et al., 2021 [59]
**Cell type classification**			
ACTINN	Immune cell	Neural Networks	Ma et al., 2020 [60]
Ikarus	Tumor cell	Logistic regression/network propagation	Dohmen et al., 2022 [61]
**Trajectory inference**			
CellRouter	Tree methods	Context likelihood of relatedness	Lummertz et al., 2018 [62]
STREAM	Graph methods	Gaussian process latent variable model	Chen et al., 2019 [63]
TinGA	Graph methods	Growing neural graph algorithm	Todorov et al., 2020 [64]
ELPIgraphy	Cyclic methods	Elastic energy functional and topological graph	Albergante et al., 2020 [65]
CStreet	Graph methods	k-nearest neighbors graph	Zhao et al., 2021 [66]
	Tree methods	Euclidean minimum spanning tree	Tenha et al., 2022 [67]

**Table 4:** Text-mining model for cancer-associated genes.

Relationships	Name	Algorithms	Author
Mutation–Gene	MuGeX	Naïve Bayes/Rocchio algorithm-TF-IDF	Erdogmus et al., 2007 [69]
Disease–Mutation		C4.5 decision tree	Singhal et al., 2016 [70]
Protein–Mutation	EnzyMiner	Probabilistic indexing	Yeniterzi et al., 2009 [71]
Variants–Literature	tmVar 2.0	Conditional random fields	Wei et al., 2018 [72]
Variant–Disease–Gene	MAGPEL	Sentence co-occurrence scoring	Saberian et al., 2020 [73]
Cancer–Genes		Hypergeometric test	Chen et al., 2021 [74]

Notes: TF: term frequency; and IDF: inverse document frequency.

**Table 5:** High-performance computing systems for cancer genome research.

Name	Computing System	Clinical Practice	Author
NVIDIA	GPUs	Mutational signature	Haradhvala et al., 2018 [77]
		Critical care	Gorzynski et al., 2022 [78]
DRAGEN	FPGAs	TSO500 FFPE pipeline	Wei et al., 2022 [79]
		TSO500 ctDNA pipeline	Pommergaard et al., 2022 [80]

Notes: GPUs: graphics processing units; FPGAs: field-programmable gate arrays; and TSO 500: TruSight Oncology 500 assay.

## Data Availability

Not applicable.
